# Tumor-Associated Mast Cells in Urothelial Bladder Cancer: Optimizing Immuno-Oncology

**DOI:** 10.3390/biomedicines9111500

**Published:** 2021-10-20

**Authors:** Hae Woong Choi, Manisha Naskar, Ho Kyung Seo, Hye Won Lee

**Affiliations:** 1 Division of Life Sciences, Korea University, Seoul 02841, Korea; haewoongchoi@korea.ac.kr (H.W.C.); manisha@korea.ac.kr (M.N.); 2 Department of Urology, Center for Urologic Cancer, National Cancer Center, Goyang 10408, Korea; 3 Division of Tumor Immunology, Department of Cancer Biomedical Science, Research Institute, Graduate School of Cancer Science and Policy, National Cancer Center, Goyang 10408, Korea

**Keywords:** bladder cancer, mast cells, mucosal immune barrier, pro-tumor immunity immunotherapy, tumor microenvironment

## Abstract

Urothelial bladder cancer (UBC) is one of the most prevalent and aggressive malignancies. Recent evidence indicates that the tumor microenvironment (TME), including a variety of immune cells, is a critical modulator of tumor initiation, progression, evolution, and treatment resistance. Mast cells (MCs) in UBC are possibly involved in tumor angiogenesis, tissue remodeling, and immunomodulation. Moreover, tumor-infiltration by MCs has been reported in early-stage UBC patients. This infiltration is linked with a favorable or unfavorable prognosis depending on the tumor type and location. Despite the discrepancy of MC function in tumor progression, MCs can modify the TME to regulate the immunity and infiltration of tumors by producing an array of mediators. Nonetheless, the precise role of MCs in UBC tumor progression and evolution remains unknown. Thus, this review discusses some critical roles of MCs in UBC. Patients with UBC are treated at both early and late stages by immunotherapeutic methods, including intravenous bacillus Calmette–Guérin instillation and immune checkpoint blockade. An understanding of the patient response and resistance mechanisms in UBC is required to unlock the complete potential of immunotherapy. Since MCs are pivotal to understand the underlying processes and predictors of therapeutic responses in UBC, our review also focuses on possible immunotherapeutic treatments that involve MCs.

## 1. Introduction

Urothelial bladder cancer (UBC) is a common disease with high morbidity and mortality rates, accounting for around 2.1% of all deaths due to cancer per year [1,2,3,4,5,6,7]. As the high rate of recurrence and the need for long-term surveillance greatly increased the economic burden of UBC patients, exploring optimized and personalized therapeutic modalities against UBCs is a rapidly evolving and expanding field in adjuvant and definitive settings [4,8,9]. Tumoral depth of invasion and detrusor invasiveness are the most significant variable for progression, recurrence, and survival in UBC [6].

At presentation, about 70% of patients with UBC present with disease confined to the mucosa (stage Ta or carcinoma in situ) or submucosa (stage T1) (non–muscle-invasive bladder cancer, NMIBC), which has a good prognosis [1,2,3,4,7,8,9,10,11,12,13]. NMIBC includes a diverse spectrum of diseases with a wide range of progression and recurrence rates that depend on several clinical and pathologic factors; thus, the key to improving the prognosis of NMIBC is to reduce the risk of recurrence and progression [3,4,7,9,11]. Standard treatment of NMIBC involves transurethral resection of the bladder tumor (TURBT), followed by intravesical chemotherapy and/or bacillus Calmette-Guerin (BCG), in a risk-adapted manner [3,4,7,9,11,12]. Especially, intravesical BCG is typically reserved for high-risk patients in the first-line setting, or as an option for intermediate-risk patients [3,4,7,8,9,11,12]. Different options exist upon failure of first-line treatment, i.e., following failure of intravesical chemotherapy or BCG, and are largely dependent on the response to prior therapy [4,9,11,12]. Following BCG failure in high-risk NMIBC, the standard of care is radical cystectomy, which is associated with considerable morbidity and mortality, particularly in older and frailer patients [3,4,7,12]. Novel therapeutic options for patients who recur after BCG are critical in order to reduce the number of patients needing cystectomy with its associated morbidity, while maintaining acceptable oncological outcomes [3,4,7,12].

By contrast, approximately 25% of bladder cancers invade the muscle layers, and 5% have metastatic disease [1,2,4,5,8,10]. MIBC has a propensity to become metastatic, and, once metastatic, it is associated with a 5-year survival of only 15% [1,10]. Current international guidelines recommend platinum-based neoadjuvant chemotherapy (NAC), followed by radical cystectomy (RC) or bladder preservation strategies, with chemoradiation (trimodality therapy) in select patients; adjuvant chemotherapy is also an option for select patients) [1,2,4,5,6,7,8,10]. The most widely used platinum-based NACs include dose-dense methotrexate, vinblastine, doxorubicin, and cisplatin (MVAC) regimens, and gemcitabine and cisplatin (GC) regimens [1,2,4,5,6,7,8,10]. Although only 5% of patients are metastatic at presentation, nearly 50% of patients with MIBC, undergoing curative-intent treatment, will eventually relapse and develop metastatic disease [2,4,5,6,7,8]. Despite high initial response rates, survival in the metastatic setting is 12–15 months with the most commonly used regimens, including GC, MVAC, or dd MVAC, but only 3–6 months if left untreated [2,4,7,8]. Unfortunately, conventional cytotoxic chemotherapy regimens have not produced optimal long-term outcomes, and approximately 30–50% of patients are not cisplatin eligible due to renal dysfunction, poor performance status, or comorbidities, such as cardiac dysfunction, neuropathy, and hearing loss [1,2,4,7,8].

Since 2016, clinically relevant benefits of immunotherapy in advanced or metastatic UBC have led to Food and Drug Administration (FDA) approval of immune checkpoint inhibitors (ICIs) as second- or first-line therapy in patients unresponsive to or ineligible for standard treatment [2,4,5,7,9]. There are two main categories of checkpoint inhibitor therapy: agents targeting programmed cell death protein 1 (PD-1) or programmed cell death-ligand 1 (PD-L1) and agents targeting cytotoxic T-lymphocyte-associated protein 4 (CTLA-4) [2,4,5,7,9]. Moreover, treatment with either antibody drug conjugates (ADCs), such as enfortumab-vedotin (EV), targets the adhesion molecule Nectin-4, or fibroblast growth factor receptor (FGFR) inhibitors, such as Erdafitinib, in patients harboring susceptible *FGFR3* gene mutations (R248Cs, S249C, G370C, Y373C) or FGFR2/3 gene fusions (FGFR3-TACC3, FGFR3 BAIAP2L1, FGFR2-BICC1, FGFR2-CASP7), and whose disease has progressed during or following platinum-based chemotherapy, with prior immunotherapy also being allowed [2,4,9,14]. Although a handful of novel agents have received FDA approval and have shown significant promise, more research is required to identify additional targets, fully elucidate their molecular mechanisms, and determine their long- term outcomes.

Mast cells (MCs) reside within the connective tissue of all vascularized organs and in mucosal tissues. They are most numerous in the skin and in the mucosal tissues of the respiratory, intestinal and urinary tracts. Their numbers and densities are highest at interfaces between the internal and external environments where they act as sentinels and can respond rapidly to foreign organisms, antigens, and toxins. Evolutionarily, MCs were probably most beneficial in rapid responses to venoms, parasites, and possibly bacterial infections. Although studies have established the role of MCs in allergies, including flushing, pruritus, urticaria, angioedema, bronchoconstriction, solid tumors sometimes associated with increased MCs on tissue biopsies include solid tumors, Hodgkin lymphoma, and skin and connective tissue tumor. Indeed, increasing evidence suggests that MCs have an enigmatic role in a variety of cancers as they either promote or inhibit tumor development depending on the conditions. UBC represents an ideal disease state to study immune evasion and mechanisms by which to improve the immune response based on several established features [8]. Although the presence of MCs in the tumor vicinity indicates that they play a role in the formation of the tumor microenvironment (TME) in UBC, their effects on tumor evolution are still debated. In the present review, we address the immunobiology of MCs in the TME of UBC. We also present the clinical applications associated with therapeutically manipulating MCs as immunogens to overcome resistance against conventional immunotherapeutic drugs.

## 2. General Immunobiology of Mast Cells

Peripherally circulating MC progenitors complete their development inside the mucosal barrier tissue where they are present near fibroblasts, blood vessels, lymphatic vessels, and neurons and lie underneath epithelial cells [15,16,17]. Expression of 47 integrins and a positive chemotaxis for the stem cell factor (SCF) recruits circulating MC from the blood to the tissues [16,18]. Subsequently, binding of the MCs to the SCF induces autophosphorylation in the MCs and activates numerous signaling molecules, including phosphatidylinositol-3-kinase (PI3K) and mitogen-activated protein kinases (MAPK) [19,20]. Additionally, MCs express various receptors, including cytokine and chemokine receptors [21], Toll-like receptors (TLRs) [22], and vascular endothelial growth factor receptors (VEGFR) [15]. Numerous other cytokines, such as interleukins (IL; IL-3, IL-4, IL-9, IL-10, IL-33) and transforming growth factor-beta (TGF-β) [15,23,24], have also been shown to affect the development and survival of MCs. Remarkably, MCs are multifaceted sentinel cells that have various immune defense and regulatory functions, including defense against venoms, parasites, and microorganisms [15,16,17]. Interestingly, MCs aid in tissue repair, matrix remodeling, fibrosis, and wound healing [25,26,27].

Numerous adhesion molecules, immune response receptors, and other surface molecules enable the MCs to respond to a wide variety of specific and nonspecific stimuli [17]. Furthermore, co-stimulatory and inhibitory molecules can potentially modify the function of the MC receptors [28,29,30]. Most significantly, MCs express FcεRI, a receptor with a very high-affinity for IgE, that stimulates the release of pro-inflammatory and immunomodulatory factors upon binding to its ligand [15,16,17,29,31,32]. Activating factors stimulate cell metabolism and induce the MCs to release pre-stored or newly formed molecules [29]. Moreover, the MCs can phagocytose, process antigens, and produce cytokines and release pre-formed mediators from their cytoplasmic granules within seconds to minutes. For instance, MCs can synthesize lipid mediators, prostaglandins, and leukotrienes de novo within minutes and can secrete a range of cytokines and chemokines post transcription and translation within hours [15,16,17,29]. These diverse biological properties of MCs and their widespread distribution and strategic proximity to blood arteries, neurons, inflamed tissues, and neoplastic foci allow them to play a critical role in multiple physiological, immunological, and pathological processes [17,33]. Nevertheless, various environmental and genetic factors regulate significant characteristics of the MCs, including proliferation, survival, and storage capacity. These factors also regulate the secretion of multiple distinct products by the MCs and regulate the intensity and nature of a response by the MCs to distinct activating signals [15,34,35,36]. Following the activation of MCs, their functional flexibility stimulates the release of compounds that have pro-inflammatory, anti-inflammatory, or immunosuppressive effects [15,37].

As tissue-resident MCs are crucial innate immune cells, their function has been highlighted in various tissue types; nevertheless, their role in the urinary bladder has received less attention [38]. In a healthy human bladder, MCs are found in the mucosa and LP [21], which is a strategic placement for optimum contact of MCs with the environment. This contact activates host defenses and induces tissue remodeling, wound healing, fibrosis, and angiogenesis [15,17,29]. Although MCs have a direct microbicidal action, they primarily function in the presence of pathogens to raise an alarm and coordinate an inflammatory response [15]. The majority of these actions may be ascribed to their role as sentinel cells and to their ability to recruit innate and adaptive immune effector cells [22,29].

## 3. Mast Cells in UBC: A Double-Edged Sword

**Figure 1:**
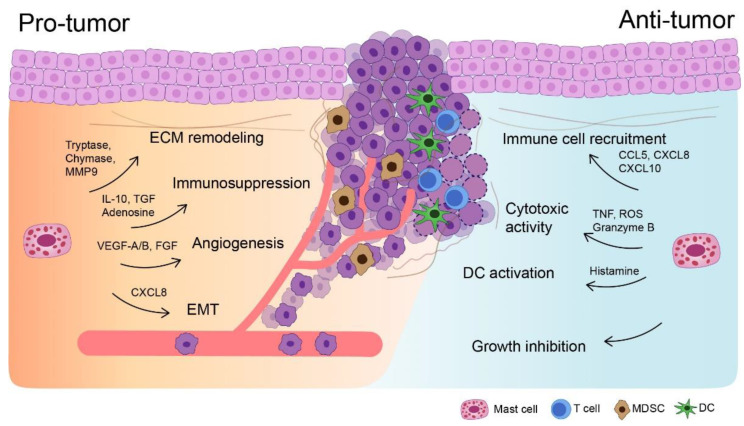
Mast cells (MCs) and their mediators have a pro- or antitumorigenic function in a variety of ways. MCs can mobilize and interact with MDSC and Treg to promote the immunosuppressive tumor microenvironment. Pro-angiogenic factors promote endothelial cell migration, proliferation, and blood vessel development. The release of proteases liberates growth factors bound to the extracellular matrix (ECM) to stimulate fibroblast proliferation and angiogenesis and that breakdown the ECM, thus facilitating tumor cell invasion. MCs can exert antitumor action through direct cyto-toxicity of tumor cells. MCs also serve as sentinels, secreting a variety of chemokines that facilitate the recruitment of anti-tumor immune effector cells to tumor locations and regulate immune effector cell responses. EMT: epithelial-to-mesenchymal transition, DC: dendritic cell.

The function of MCs in many tumor types is complex and remains controversial [19], as they either promote or inhibit tumor growth, depending on the type and stage of cancer and their localization in the TME (Figure 1) [15,16,17,19]. Activated MCs may selectively produce pro- and anti-inflammatory chemicals, and their phenotypes may alter in response to the TME, thereby making them analogous to a “double-edged sword” [15,19]. For example, MCs that secrete IL-1, IL-4, IL-6, and tumor necrosis factor (TNF)-α can actively eliminate tumor cells and halt tumorigenesis [25,39]. On the contrary, it is well established that various subsets of MCs infiltrate tumors at various phases of progression; the MC count is related to the stage, prognosis, and invasiveness of the tumor [16,17,19]. The mechanism by which MCs promote tumor growth is very complex and involves tissue remodeling, angiogenesis, and immune modulation [40,41]. The existence of a potential connection between MCs, chronic inflammation, and cancer has been proposed for an extended period [29]. In chronic inflammation, the tissue interstitium is associated with oxidative stress, edema, enzymatic stress, persistent leukocyte stimulation, lymphangiogenesis, angiogenesis, and fibrosis [19,42,43,44,45]. Chronic inflammation induces angiogenesis directly and promotes tumor growth and immune suppression [29]. Additionally, the involvement of a noxious factor can promote the participation of MCs in the chronicity of an inflammatory response [19,24,46,47].

## 4. Evidence for Pro-Tumorigenic Roles of Mast Cells in Urothelial Bladder Cancer

**Figure 2:**
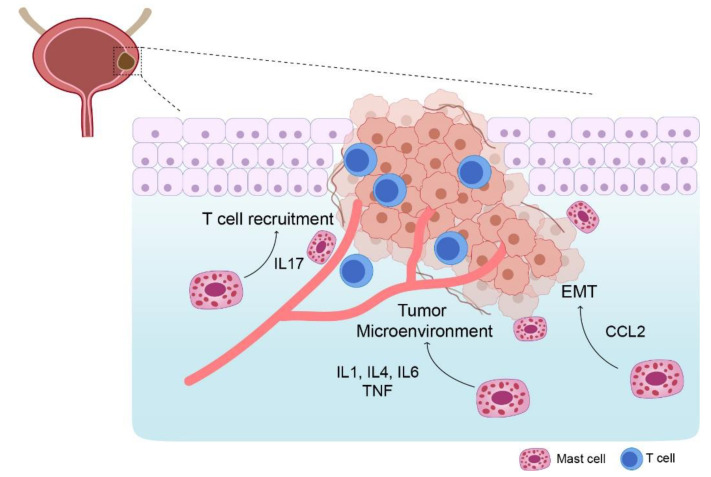
Modulation of bladder cancer by mast cells. Activated MCs can amplify the tumor microenvironment’s dysregulated tissue homeostasis and promote tumor development. MCs in the tumor microenvironment enhance epithelial-mesenchymal transition induction. Mast cells contribute anti-tumor immunity by mobilizing and activating immune cells.

Interactions between MCs and bladder tumor cells may result in the activation of MCs and release of mediators (Figure 2) [15,25,48]. Upon activation, MCs produce multiple growth factors, angiogenic factors, and pro-inflammatory chemicals that contribute to the aggressive phenotypes of tumor cells [15,25,48]. Moreover, MCs infiltrate tumors and promote their proliferation and invasion [49]. Their recruitment to the tumors increases the interaction between estrogen receptors (ERs) and C–C Motif Chemokine Ligand 2 (CCL2), wherein CCL2 promotes epithelial-to-mesenchymal transition (EMT) and the production of matrix metalloproteinases (MMP) in the tumor location. Therefore, this indicates that the activation of the ERβ/CCL2/EMT/MMP axis by the MCs increases UBC invasion [49]. MCs operate by increasing the motility, proliferation, and differentiation of endothelial cells and promote tumor-endothelial cell adhesion [25]. Analysis of the UBC tumor tissues revealed a strong association between the microvessel density and the number of MCs present in the tumoral zone [50]. Numerous angiogenic mediators are produced by the MCs in the TME, including OX40L, VEGF, IL-8, nerve growth factor, TNF-α, TGF-β, tryptase, histamine, CXCL12, CXCL8, urokinase-type plasminogen activator, prostaglandin E2 (PGE2), platelet-derived growth factor-β, fibroblast growth factor-2 (FGF-2), IL-6, IL-8, and thymidine phosphorylase [15,16,17,19,25,41,51,52,53]. Notably, the overall function of MCs in tumor angiogenesis is dependent on the stimuli that activates them and the subsequent mediator produced by them [25,54].

Tumor-infiltrating MCs directly affect the aggressiveness of a tumor cell. However, they indirectly function inside the TME by interacting with other immune cells and promoting or suppressing immunological responses [15,25,40]. Since the MCs interact with immune suppressor cells, such as MDSCs and Tregs, they can substantially influence the development and functional capability of tumor immunity [15,19,55,56,57,58,59,60,61]. For example, MDSCs are recruited to tumor locations by CCL2 and potentially CXCL1 and CXCL2, where direct contact with MCs or with histamine increases their inhibitory function [19,55]. The MC stimulates the production of IL-17 via MDSC, mobilizing Tregs and enhancing their suppressive activity on cytotoxic T cell [19,57]. Subsequently, IL-17 indirectly recruits Tregs and increases their suppressor function and stimulates IL-9 production, thus enhancing the pro-tumorigenic function of the MCs in the TME [19,57]. Remarkably, TGF-β and IL-10 produced by the MCs promote the development of Tregs, downregulate expression of costimulatory molecules in DCs, and reduce the production of pro-inflammatory cytokines by TAMs. They also suppress antigen-specific T cell responses and enhance fibrosis [55,58,59,60]. The presence of more tumor-specific IFN-γ^+^ CD8^+^ T cells in the tumor-draining lymph nodes of MC-deficient mice than that in wild-type mice supports the notion that MCs suppress tumor-specific T cell response in UBC [55,56].

## 5. Cytokine Milieu in the Tumor Microenvironment Generated by Mast Cells

Histamine, a primary mediator secreted by MCs, exhibits either tumorigenic or antitumor downstream effects depending on the TME and its receptors H1R, H2R, H3R, or H4R [25,55,62,63,64,65]. Depending on which receptor is activated, histamine may promote the function of particular Th subtypes or Treg responses and alter immunophenotype of monocytes; thus, immunosuppressive signals to NK cells are downregulated [55,63,64,65]. Tryptase, a serine protease produced during MC activation, stimulates angiogenesis and ECM breakdown, resulting in tumor progression and metastasis [25,38,55,62]. Based on these findings, three MC tryptase inhibitors, nafamostat mesylate, tranilast, and gabexate mesylate, have been demonstrated as anti-cancer agents in multiple solid tumor types either in conjunction with other cancer treatments or as an individual treatment [55,66,67,68]. Although tryptase inhibitors reduce angiogenesis and activation of MMPs, they also exhibit other activities, such as suppression of TGF-β, inhibition of other proteases, downregulation of NF-κB, and inhibition of the MAPK signaling pathway [55,69].

MCs release chymase and leukocyte elastase during inflammatory processes and degranulation. These molecules act on matrix-associated latent TGF-β complexes, releasing the latent TGF-β from the subendothelial ECM [15,41]. TGF-β acts a tumor suppressor during the premalignant phases of tumorigenesis, but it promotes tumor growth in the later stages, resulting in metastasis [19,70]. Additionally, TGF-β can directly suppress the immunological functions of effector T cells, NK cells, and B cells [41,71].

## 6. Mast Cells Boost Therapeutic Efficacy against Non-Muscle Invasive Bladder Cancer

As previously mentioned, NMIBC are characterized by frequent recurrence and progression to muscle invasive bladder cancer (MIBC) [1,2,3,4,7,8,9,10,11,12,13,72]. Transurethral resection of bladder tumors, followed by intravesical Bacillus Calmette–Guérin (BCG) instillation, is the current gold standard for treating patients with intermediate- and high-risk NMIBC [1,2,3,4,7,8,9,10,11,12,13,72,73,74,75,76,77,78]. The BCG vaccine, comprising live attenuated *Mycobacterium bovis,* binds to fibronectin in the urothelium and causes a direct tumor and immunological response [72,79]. It has been utilized as immunotherapeutic agent for NMIBC for over 40 years; however, the mechanism underlying a BCG-mediated anti-tumor response is not clearly elucidated [72,75]. Nevertheless, this mechanism is likely a complex interaction between the innate and adaptive immune system [26,27,75,80]. Remarkably, BCG stimulates function/activity of APCs, activates the innate immune system, induces DC maturation, and upregulates CD83, CD80, and CD86 markers and IL-12 and TNF-α levels [26,27,80]. In addition, BCG induces the conversion of monocytes to active macrophages and is responsible for the ultimate maturation of myeloid cells [26,27]. Notably, the numbers of TAMs and DCs present in a patient before intravesical BCG instillation is associated with cancer recurrence [75,81,82]. TAMs are associated with a higher risk of recurrence upon BCG therapy [75]. Additionally, analyses of DCs exposed to BCG demonstrated the activation of NK cells, NK T cells (NKT), and γδ T cells and the cytolytic killing of BCG-infected tumor cells in the bladder [75,83,84,85,86,87]. While infiltration of CD4^+^ T cells and T-bet^+^ T cells in tumors prior to BCG therapy is associated with lower cancer recurrence in patients, that of Tregs and a reduction in numbers of GATA3^+^ Th2 cells is associated with higher recurrence. Thus, a patient’s response to intravesical BCG partly rests on a balance between the functions of Th1, Th2, and Th17 [75,84].

A clinical study demonstrated that the number of IL-17-positive MCs increased in few patients with carcinoma in situ while they underwent BCG therapy [88]. It was speculated that this occurrence was because of the release of IL-17 by the MCs in response to the BCG treatment. Subsequently, the MCs stimulate IL-8 production in the urothelium [88]. Further, it was hypothesized that neutrophils and various other immune cells were thereafter chemotactically attracted to the tumor site, thus amplifying the overall immune response [88]. This study can help stratify the cases that can have better survival and respond much better to the BCG treatment [72,89].

## 7. Manipulating Mast Cells to Boost Treatment-Induced Immunogenic Cell Death

Increased tumor-infiltration by MCs prior to surgery is significantly associated with poor response to pre-surgical chemotherapy in the aggressive form of locally advanced solid tumors [16,55]. Moreover, recent data indicate that increased tumor-infiltration by MCs predicts a poor response to ICIs that target PD-1 in melanoma [55,90]. Notably, increased infiltration of tumors by stromal MCs is an independent prognostic marker that indicates an unfavorable prognoses for patients with MIBC [91]. A retrospective investigation evaluating the effectiveness of adjuvant chemotherapy in patients with MIBC discovered that individuals who had minimal tumor-infiltrating MCs have a low risk of mortality and cancer recurrence [91]. Furthermore, differential gene expression profiles in MIBC specimens revealed that bladder tumors with a low number of invading MCs expressed more genes associated with immune activation [91]. Spatial analysis revealed close proximity between CTLs and the MCs, highlighting MCs as promising therapeutic targets that can improve current therapeutic strategies against UBC [16].

Most importantly, existing literature indicates that MCs play a critical role in orchestrating an initial antitumor immune response but may also be responsible for inducing resistance against ICIs [55]. Tumor RNA-seq, multiplexed imaging, and immunohistochemistry labeling have shown elevated chemokine expression, particularly with the recruitment of MCs and FOXP3^+^ Tregs at selected tumor locations [90]. These tumor-invading cells are linked with decreased expression of HLA-class I molecules on the tumor cells, a deficiency of CD8^+^ T cells in tumor locations, and efficient killing of tumor cells and eventual immunological escape following anti-PD-1 treatment [90]. When anti-PD-1 is combined with sunitinib or imatinib, MC numbers are depleted, and tumors completely regress, suggesting that MC depletion may improve the effectiveness of anti-PD-1 treatment [90]. Moreover, inhibition of MC-associated PD-L1 improved tumor control and boosted tumor-infiltration by CD3^+^ T-cells and elevated IFN-γ and granzyme B production [55].

**Figure 3:**
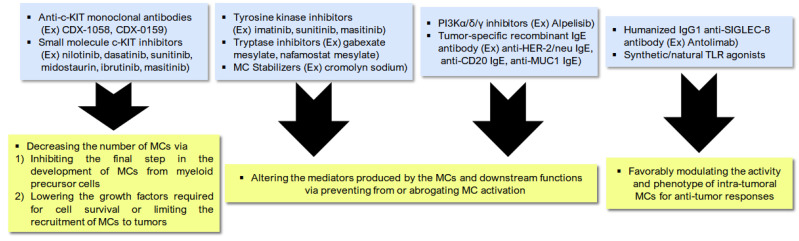
Potential therapeutic strategies targeting tumor-infiltrating MCs to promote anti-tumor immunity.

Resistance to anti-PD-1 therapy mediated by the MCs requires greater research to discover new treatment avenues [90]. As the MCs are associated with resistance to anti-PD-1 treatment, their depletion enhances patient response for anti-PD-1 therapy [55,90]. Generally, therapeutics directed against the MCs in cancer have one of the three mechanisms of action: decreasing the number of MCs, modifying MC activity and phenotype, and altering the mediators produced by the MCs and their downstream functions [55]. c-KIT inhibitors, MC stabilizers, FcεR1 signaling pathway activators/inhibitors, antibodies against inhibitory receptors and ligands, and TLR agonists and modulators of MC mediators are all possible treatment approaches (Figure 3) [55].

Reduced MC numbers can be achieved by inhibiting the final step in the development of MCs from myeloid precursor cells. Other alternatives include lowering the growth factors required for cell survival or limiting the recruitment of MCs to tumors [55]. The SCF is a cytokine that binds to the c-KIT receptor and is critical for MC differentiation, survival, proliferation. Thus, MCs can be targeted pharmacologically by small molecule inhibitors targeting c-KIT used in clinical practice, including nilotinib, dasatinib, sunitinib, midostaurin, ibrutinib, and masitinib [55,92]. Monoclonal antibodies targeting c-KIT, such as CDX-1058 and CDX-0159, are in clinical development for inflammatory disease and c-KIT-positive solid tumors (NCT02642016) and have a greater specificity for the intended target than the TKIs [55]. However, clinical translation is hampered by the plasticity and context-based functions of the MCs [55]. Therefore, it is critical to include patient sample-based translational research into an investigation on the biologic relevance and therapeutic efficacy of MC-directed treatments [55].

Another therapeutic strategy for targeting the MCs is the prevention or abrogation of MC activation [25,55,93,94]. Targeting MC activation by inhibiting the secretion of mediators using tyrosine kinase inhibitors (TKIs; imatinib, sunitinib, and masitinib) or using tryptase inhibitors (gabexate mesylate and nafamostat mesylate) can be beneficial as an anti-cancer therapy [25,55,90,95,96]. Furthermore, stabilizing medications, such as cromolyn sodium, are often used in allergy disorders to inhibit degranulation in MCs and have been studied in various preclinical solid tumor models [25,55,93,94]. For instance, in a study on MYC-induced pancreatic neuroendocrine tumors, association of MYC activation with MC recruitment was observed to be necessary for tumor growth, and treatment with cromolyn sodium inhibited MC degranulation and reduced tumor growth [55,93].

Alternatively, intracellular upstream signaling pathways within MCs can also be targeted [55]. Binding of IgE to FcεR1 causes FcεR1 aggregation, following which downstream immunoreceptor tyrosine kinases are phosphorylated and activated. Subsequently, the spleen tyrosine kinase (SYK), PI3K, and Bruton’s tyrosine kinase (BTK) are activated, inducing the secretion of inflammatory mediators [55,97,98,99]. This signaling cascade can be blocked downstream of IgE binding to FcεR1 by inhibiting the activation of SYK, PI3K, and BTK [55,97,98]. Inhibitors of PI3Kδ and PI3Kγ are presently undergoing clinical trials for various malignancies and, most notably, for combination immunotherapy [55,97,98]. Alpelisib, an inhibitor targeting PI3Kα, has been investigated in allergic rhinitis and is now being utilized in ER^+^ positive metastatic breast cancer [55,97,98].

Since MCs and MC-induced inflammation induce anti-tumor responses, studies have proposed the use of anti-tumor IgE antibodies for cancer immunotherapy [55,100,101,102]. The high density of FcεR1 and the prolonged half-life of antibodies makes this an appealing treatment approach, particularly for malignancies with high MC infiltration. Omalizumab, a humanized monoclonal antibody directed against IgE has been demonstrated to be effective in severe allergic asthma and is often administered to patients today. In vitro experiments using humanized monoclonal anti-HER-2/neu IgE and humanized anti-CD20 IgE targeted MC degranulation and reduced tumor cell proliferation [55,101]. Combining anti-MUC-1 IgE with chemokines that target the MCs in an MUC-1-expressing 4T1 murine breast cancer model resulted in tumor rejection. Importantly, the 4T1 cells were also subsequently rejected on the contralateral flank in the absence of either the IgE antibody or chemokines, suggesting the activation of a memory immune response [55,100]. Of note, anti-tumor IgE antibodies are limited to targetable tumor antigens, such as HER2, CD20, and MUC-1; thus, FGFR 2/3 and necti-4 in UBC are potential tumor-specific targets [55].

Given the abundance and plasticity of the MCs, there have been considerable attempts to favorably modify the function of pre-existing intra-tumoral MCs to induce an anti-tumor response rather than to deplete MC levels [55,103,104]. Targeting TLRs is a feasible therapeutic approach for doing this, either by direct inhibition with synthetic TLR agonists or indirect inhibition with natural TLR agonists produced as intermediates in response to other immunotherapies [58,103,104]. Conventionally, cancer immunotherapy targeting TLRs has focused on increasing TLR activity in macrophages, DCs, and B cells. Presently, the critical role of TLRs on monocytes and macrophages in defining cancer immunity has been gaining momentum [58,103,104]. Furthermore, antibodies directed against inhibitory cell surface receptors to suppress MC activation are another avenue of active research [58]. Recently, SIGLEC-8 was discovered as an inhibitory receptor that is mostly expressed on the surface of the MCs, eosinophils, and, to a lesser degree, basophils [103,104]. When bound to its ligand, it directly induces antibody-dependent cell-mediated cytotoxicity and decreases degranulation in the MCs [58]. Remarkably, antolimab is a humanized IgG1 monoclonal antibody that inhibits SIGLEC-8 and, therefore, decreases MC activation and inflammation in anaphylaxis mice models [58].

## 8. Take-Home Messages and Challenges

Therapeutic strategies that disrupt the function of the MCs or that of their mediators are common [29]. Notably, the MCs can serve as a novel target for adjuvant therapy for cancers, as they can selectively inhibit angiogenesis, tissue remodeling, and tumor-promoting molecules, thus secreting cytotoxic cytokines and preventing MC-mediated immune suppression [29]. Pre-clinical studies in experimental models, using anti-c-KIT antibodies [105], anti-TNFα antibodies [106], or the MC stabilizer cromolyn [93], have shown promising results [29]. The c-KIT pathway is often activated in tumors, identifying c-Kit as a proto-oncogene. As a result, targeting of the c-KIT pathway was considered to be an optimal approach for a tumor-specific treatment [29]. Consequently, novel TKIs may be effective against wild type c-KIT, which is expressed by the tumor-infiltrating MCs, and may be beneficial in eliminating the MCs. Additionally, c-KIT-targeted therapy with TKIs may ideally work against both tumor and stromal cells [29]. Despite seeking to positively regulate the anti-tumor capabilities of the MCs, clinical evaluation of toxicities is necessary to ensure that critical biological responses necessary for the health of the host are not negatively affected [55].

Our understanding of the MC immunobiology in cancer is limited because majority of the research has been conducted in vitro using human and murine samples, as the MC biology differs inherently in both these samples. Moreover, it is difficult to decipher the specificity of the MC mediators, such as cytokines and chemokines, as they are secreted by multiple cell types [55]. The function of an MC depends on factors, such as the cancer type and stage, likely treatment history, concurrent anti-cancer therapies, and on the activation and location of MCs within a tumor and how they are altered by the therapeutics being investigated [55]. Advances in immune monitoring provide in-depth profiling data on immune cells and include techniques, such as single cell sequencing technologies, functional assays to assess polyfunctional responses, and multiplexed immunohistochemistry to determine spatial organization and interaction of the cells [55]. Studying the pharmacodynamic changes in intratumoral MCs and mediators in patient samples subjected to these therapies and studying the role of MCs specifically in these therapies in pre-clinical studies can provide an added insight into cancer therapy targeting the MCs [55]. Thus, discovering therapeutically relevant characteristics and mechanisms in the MCs and understanding the longitudinal changes in these features and pathways in response to systemic therapies is critical to determine an optimal therapeutic approach [55]. Nevertheless, preclinical studies, such as combination trials involving PD-1 immune checkpoint inhibition in multiple malignancies, indicate that discovery of optimum treatment combinations may be more necessary than monotherapy involving MCs in UBC [55]. Notably, it is critical to combine rational trial design with pharmacodynamic evaluations to discover the most effective treatment responses and possible resistance mechanisms when designing clinical trials to evaluate novel combinations [55]. The timing and agent selection are critical factors in a combination therapy [107].

As one of the earliest cells to infiltrate tumors, MCs are crucial components of the immune system that can induce angiogenesis and aid tumor progression. Opposingly, the MCs are beneficial to patients as they selectively recruit various immune cells to the tumor region. Their role in the development and progression of UBC needs a detailed investigation. Moreover, their role may vary depending on the stage and invasiveness of the cancer. Thus, further investigation into the recruitment of MCs to tumors and the functional role of MC mediators can delineate the potential of MCs as novel immunotherapeutic targets against UBC. Furthermore, interaction of the MCs with other cell types in the TME should be studied further to elucidate all possible interactions and prognostic implications. Focusing on the activation of MCs and the release of inflammatory cytokines in UBC may help develop innovative UBC-control strategies. Moreover, study on the architecture and geographic distribution of MCs can provide further insights into their involvement in UBC biology. Profiling the heterogeneity of MCs in benign and malignant solid tumors can target and avoid MC-mediated tumor angiogenesis. Additionally, particular attention must be paid to determine the composition of MCs in the TME in UBC, since contact with the complex tumor environment has been demonstrated to affect the functional expression of various membrane receptors. To monitor the onset of ICD and its subsequent consequences on MC immunobiology in UBCs, clinical studies must regularly integrate a strong biomarker strategy [108]. These biomarkers must assess the type of cell death and quantify the release of DAMPs, as well as determine the number, identity, and location of immune cells involved in a functioning adaptive immune response [108]. Biomarkers should distinguish between natural and therapeutically-mediated ICD by focusing on cell types that are most sensitive to DAMPs [108]. Since the end stage of an ICD is a protective T cell response, T cell numbers should also be quantified to determine the effectiveness of treatment strategies [108].

## Data Availability

This article does not report any data.
